# The effectiveness of ramosetron and ondansetron for preventing postoperative nausea and vomiting after arthroscopic rotator cuff repair: a randomized controlled trial

**DOI:** 10.1186/s13018-020-02060-3

**Published:** 2020-11-11

**Authors:** Sang-Uk Lee, Hyo-Jin Lee, Yang-Soo Kim

**Affiliations:** 1 Department of Orthopedic Surgery, Incheon St. Mary’s Hospital, College of Medicine, The Catholic University of Korea, Incheon, Republic of Korea; 2 Department of Orthopedic Surgery, Seoul St. Mary’s Hospital, College of Medicine, The Catholic University of Korea, 222, Banpo-daero, Seocho-gu, Seoul, Republic of Korea

**Keywords:** PONV (postoperative nausea and vomiting), Rotator cuff tear, Arthroscopic cuff tear repair, Ramosetron, Ondansetron

## Abstract

**Background:**

Arthroscopic rotator cuff repair is a painful procedure, and treatment of emetic events associated with drugs used in the current multimodal pain management remains challenging. This study aimed to evaluate the effectiveness of ramosetron or ondansetron to relieve postoperative nausea and vomiting (PONV) and pain after arthroscopic rotator cuff repair.

**Methods:**

In total, 122 consecutive patients undergoing arthroscopic rotator cuff repair were randomly allocated into three groups: ramosetron group (*n* = 39), ondansetron group (*n* = 43), and control group (*n* = 40). Then, 0.3 mg of ramosetron or 8 mg of ondansetron was administered intravenously at the end of surgery according to group. All patients received general anesthesia and multimodal pain management protocol including preemptive analgesic medication, fentanyl-based intravenous patient-controlled analgesia, and postoperative analgesic medication. Incidence of emetic events, rescue antiemetic requirements (10 mg of metoclopramide, IV), complete response, pain level, and side effects were recorded in three periods: 0–6, 6–24, and 24–48 h postoperatively. The severity of nausea and pain was evaluated using a visual analog scale.

**Results:**

The ramosetron group tended to have a lower incidence and severity of nausea during the 6- to 24-h postoperative period and fewer rescue antiemetic drug requirements during the 0- to 48-h period than the control group, showing statistical significance. Additionally, the frequency of complete response of the ramosetron and ondansetron groups was significantly higher than that of the control group. No difference was found among the groups in the pain level except during the 0- to 6-h period. The two groups have a higher complete response during the 6- to 24-h period than the control group.

**Conclusions:**

Ramosetron use led to a lower incidence, mild severity of nausea, and reduced use of rescue antiemetic drug after arthroscopic rotator cuff repair during the 6- to 24-h postoperative period than the control.

**Level of evidence:**

Level I, randomized controlled trials, treatment study

## Introduction

Rotator cuff disease is one of the common causes of shoulder pain and is commonly treated by arthroscopic rotator cuff repair, in which patients tend to experience intense postoperative pain [1, 2]. Adequate pain management would not only increase patient satisfaction, but also shorten the hospital stay [3, 4]. However, many anesthetic and analgesic drugs used in pain control commonly provoke postoperative nausea and vomiting (PONV) after orthopedic surgery, from 20 to 81% [5–8]. Although intravenous (IV) opioids have many advantages in the pain management, PONV causes dehydration, increased pain perception, wound dehiscence, delayed recovery, worsened patient satisfaction [9, 10], and pulmonary aspiration [11]. Therefore, when patients with high risk of PONV are planned to be treated with opioid-based IV patient-controlled analgesia (PCA), appropriate prophylactic antiemetic treatment should be considered, rather than treating the established PONV.

Several studies report variable responses to specific antiemetic drugs [12–14]. Among various antiemetic drugs tried, serotonin receptor antagonists such as ondansetron [15], granisetron [16], and dolasetron [17] are the most commonly used to prevent PONV. However, they have very short duration of action to cover the immediate postoperative period and have limited effect on postoperative vomiting rather than an anti-nausea action [9, 18, 19]. Several studies reported that ramosetron, which is a serotonin 5-hydroxytryptamine type 3 (5-HT3) receptor antagonist for PONV treatment, has better effectiveness and longer-acting properties than other serotonin receptor antagonists [6, 9, 20].

However, information on the effectiveness of ramosetron on PONV prevention in orthopedic patients is limited, and a few clinical studies have compared the prophylactic efficacies of ondansetron, ramosetron, and placebo after arthroscopic rotator cuff repair. Therefore, the aim of this prospective, randomized, double-blinded trial was to compare the antiemetic effectiveness of prophylactic administration of ondansetron, ramosetron, and placebo in high-risk patients with fentanyl-based PCA after arthroscopic rotator cuff repair. We hypothesized that [3] patients receiving ramosetron or ondansetron medications after arthroscopic rotator cuff repair would have less postoperative emetic events in the early postoperative period than the control groups, [21] ramosetron or ondansetron reduces postoperative emetic events and the use of rescue antiemetic drug, and [22] ramosetron or ondansetron influences pain levels in patients managed with analgesics and fentanyl-based intravenous PCA after arthroscopic rotator cuff repair.

This article fits into the framework of translational orthopedic: how to evaluate the efficacy of ramosetron in surgery of arthroscopic rotator [23–25].

## Materials and methods

### Patient population

This prospective randomized, double-blinded trial study was approved by the Hospital Institutional Review Board, and informed written consent was obtained from all reviewed subjects. However, in 2011, when we conducted the study, the Clinical Research Information Service (CRIS) was not implemented in our country. So, we enrolled in CRIS retrospectively for our experiments and then were issued the registration number (KCT0004460 on CRIS). The authors confirm that all ongoing and related trials for this drug/intervention are registered.

A total of 122 patients undergoing arthroscopic rotator cuff repair surgery between September 2011 and February 2013 were randomized to receive either ramosetron (*n* = 39), ondansetron (*n* = 43), or placebo (*n* = 40). So, we enrolled and followed up patients from September 1, 2011, to February 31, 2013.

The inclusion criterion was ambulatory patients undergoing arthroscopic rotator cuff repair surgery. The exclusion criteria were as follows: (a) previous surgery, (b) trauma history, (c) intolerance or allergy to any drugs used in the study, (d) severe bowel motility impairment, (e) administration of another antiemetic drug 24 h before surgery, (f) alcohol or opioid dependence, (g) history of cardiovascular or respiratory disease, and (h) renal or hepatic functional impairments.

**Fig. 1:**
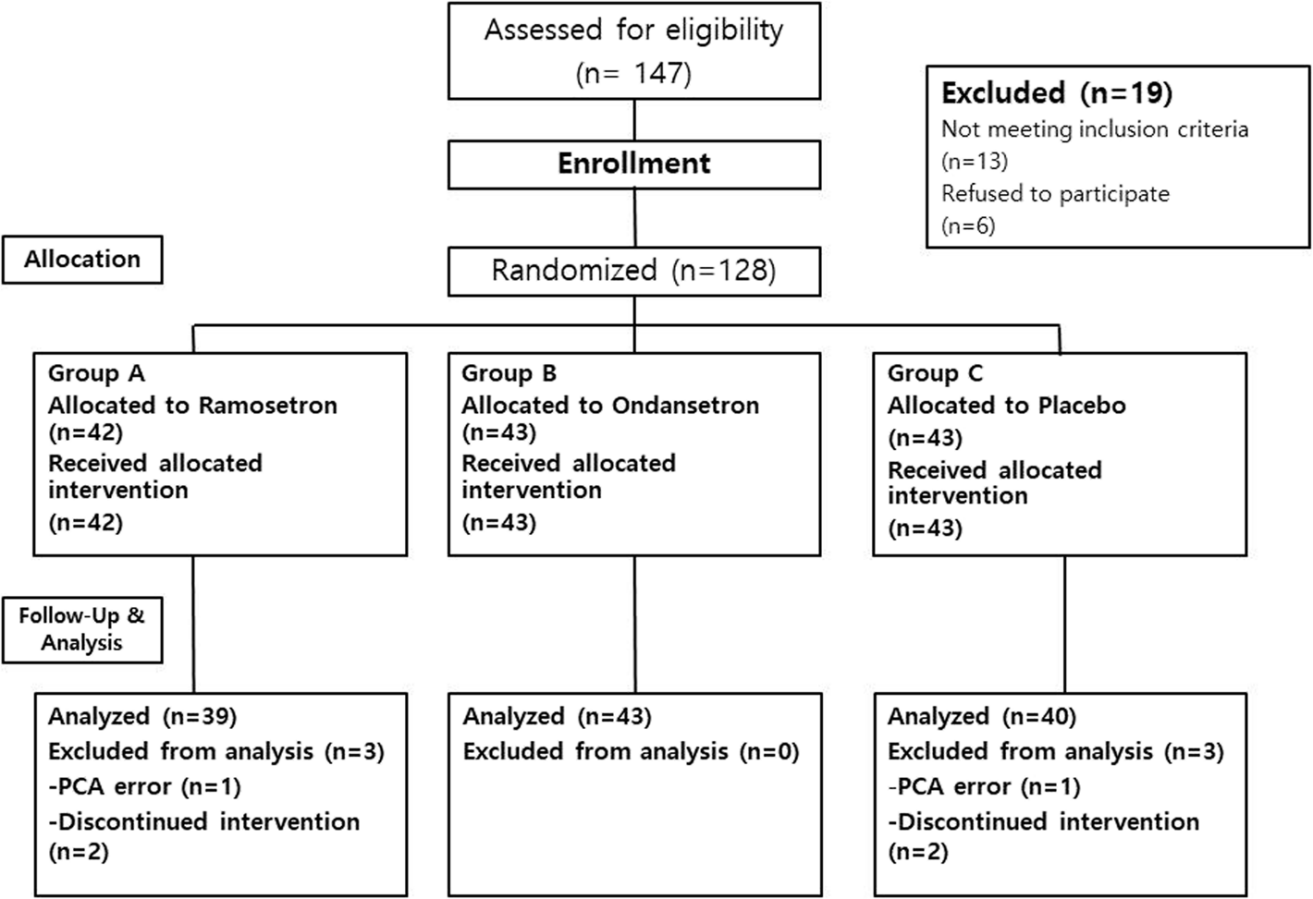
CONSORT flow diagram. PCA, patient-controlled analgesia

In addition, we excluded patients when general anesthesia was contraindicated. After assessing 147 patients for eligibility, we excluded 19 patients before enrollment for various reasons; subsequently, 128 patients were enrolled for randomization (Fig. 1). Patients were randomly allocated into three groups by a computer-generated randomization table (Random Allocation Software Version 1.0). Patients were allocated into three groups: ramosetron group, ondansetron group, or control group (normal saline IV).

Initially, 42 patients were allocated to the ramosetron group and 43 to the ondansetron and control groups respectively. We excluded three patients in the ramosetron group and three in the control group according to the defined exclusion criteria, leaving 122 patients (ramosetron group, *n* = 39; ondansetron group, *n* = 43; control group, *n* = 40) for analysis.

### Routine pre- and postoperative care and data collection

All patients received the same anesthetic and multimodal pain management protocol, except that ramosetron 0.3 mg in 2 mL or ondansetron 8 mg in 2 mL or normal saline 2 mL was administered intravenously at the end of surgery according to group. Briefly, oral analgesic drugs (10 mg oxycodone, 200 mg of celecoxib, 75 mg of pregabalin, and 650 mg of acetaminophen) were administered for preoperative preemptive analgesia on a call basis to all 122 patients before surgery. Anesthesia was induced with 1.5 to 2.5 mg/kg of propofol, 0.5 to 1.5 μg/kg of remifentanil, and 0.6 mg/kg of rocuronium and maintained with 1.5 to 2.5% of sevoflurane (50:50 mixture of oxygen and air) and 0.1 to 0.3 μg/kg/min of remifentanil. Then, 2 mL of medication containing either 0.3 mg of ramosetron (Nasea; Astellas, Tokyo, Japan) or 8.0 mg of ondansetron (Zofran; GlaxoSmithKline, Parma, Italy) or normal saline 2 mL was injected 10 min before the end of surgery. Then, IV-PCA opioid was introduced. The IV PCA regimen was comprised of a mixture of 2 mg of fentanyl and 120 mg of ketorolac and normal saline in a total volume of 100 mL. The IV PCA maintenance dose was allowed only at 1 mL/h, respectively. To avoid the bias of time-dependent different dose, bolus dose injection was not permitted.

Patients were administered every 12 h for 3 days after surgery with the following medications: 200 mg of celecoxib, 75 mg of pregabalin, and 650 mg of acetaminophen. Rescue antiemetic (10 mg of metoclopramide, IV) or analgesic (100 mg of ketoprofen, IM) was administered according to the decision of blinded orthopedic physicians in charge of a patient in wards or upon patient’s request.

Incidence of PONV and severities of nausea were recorded during three postoperative periods (0–6, 6–24, and 24–48 h). Nausea was defined as a subjective unpleasant sensation associated with the awareness of urge to vomit and vomiting as the forceful expulsion of gastric contents from the mouth [26]. The severity of nausea was assessed by patients using a 0 to 10 VAS (the left end “0”corresponded to no nausea and the right end “10”to the worst imaginable nausea). Other outcome variables were number of required rescue antiemetics, whether a complete response to the administered rescue antiemetics was achieved, pain level, and side effects. Complete response to an administered rescue antiemetic was defined as no additional experience of PONV without the requirement for another rescue antiemetic [27]. Pain levels were also estimated using VAS that ranged from 0 (no pain) to 10 (worst imaginable pain) for the three periods.

### Statistical analysis

We compared the ramosetron, ondansetron, and control groups with respect to outcomes. Sample size was calculated with reference to the results of a study comparing the effects of ramosetron and ondansetron on PONV associated with IV-PCA use in highly susceptible patients [28]. We calculated that the inclusion of 39 patients per group would afford an 80% chance of detection of a 20% reduction in the incidence of PONV using the Fisher’s exact test with a type I error of 0.05. All statistical analyses were performed using SPSS version 18.0 (SPSS Inc., Chicago, IL, USA). The Shapiro-Wilk test and Kolmogorov-Smirnov test were used to ensure normally distributed data. Continuous variables (age, weight, body mass index, duration of anesthesia, severity of nausea, and pain score) were analyzed by analysis of variance, and intergroup differences in nonparametric variables were compared using the Kruskal-Wallis test and Mann-Whitney test. Categorical variables (sex, motion sickness, PONV history, smoking, PONV incidence, requirements for rescue antiemetics, proportion of complete response to the administered rescue antiemetics, rescue analgesics, and adverse events) were compared using the chi-squared test or Fisher’s exact test. Data are expressed as means ± standard deviation (SD) or counts (%). A *p* value < 0.05 was considered statistically significant.

## Results

The preoperative characteristics and operative data were similar in the three groups (Table 1). During the 0- to 6-h period after surgery, the overall number of patients who experienced postoperative nausea among all groups was relatively high compared with that in the other period (Table 2): 16 patients in the ramosetron group, 16 in the ondansetron group, and 18 in the control group. However, there were no significant differences among the groups. The number of nausea-free patients was greater in the ramosetron group than in the control group during the 6- to 24-h period (*p* = 0.007) (Table 2). Prophylactic use of ramosetron tended to reduce the severity of nausea (*p* = 0.003) during the 6- to 24-h period. However, it showed no statistical difference compared with the ondansetron group even though incidence and severity of nausea in ramosetron group is lower (13% versus 28%, VAS 0.4 ± 1.3 versus VAS 1.1 ± 2.5) than the ondansetron group (Tables 2 and 3). Additionally, no differences were noticed among the three groups during the other two periods. Prophylactic use of ramosetron and ondansetron improves the rate of complete response than the control during the 6- to 24-h period (*p* = 0.005) (Table 4). The severity of pain was lower in ondansetron group than in the other two groups during the 0- to 6-h period (*p* = 0.001), but no differences were also found among three groups during the other two periods (Table 5). The overall incidence of vomiting, rescue antiemetic requirement, and rescue pain killer requirement were similar among the three groups during the three periods (Tables 2, 4, and 5). However, the ramosetron group tended to have fewer rescue antiemetic drug requirements than the control group during the 0- to 48-h period (Table 4). The three groups were comparable in terms of the number of patients who experienced adverse events postoperatively except headache during the 0- to 6-h period (Table 6). The incidence of headache was higher in ramosetron group and ondansetron than in the control group during 0–6 h.

**Table 1:** Demographics in the three groups

	Ramosetron (***n*** = 39)	Ondansetron (***n*** = 43)	Control (***n*** = 40)	***p*** value
Age (year)	61.4 ± 9.7	63.8 ± 8.1	59.2 ± 10.2	0.087
Gender (M/F)	21/18	14/29	17/23	0.150
Weight (kg)	64.8 ± 10.8	60.8 ± 8.5	63.1 ± 11.7	0.211
BMI (kg/m²)	24.1 ± 3.2	23.9 ± 2.8	24.1 ± 3.5	0.943
Anesthesia time (min)	138.3 ± 72.6	120.7 ± 24.6	130.9 ± 27.0	0.152
Motion sickness	0	3	2	0.370
History of PONV	1	2	3	0.688
Smoking status	5 (13)	3 (7)	8 (20)	0.213

Values are mean ± SD or numbers of patients (percentage)

*PONV* postoperative nausea and vomiting, *BMI* body mass index, *SD* standard deviation

**Table 2:** Incidences of PONV in the three groups

	Ramosetron (*n* = 39)	Ondansetron (*n* = 43)	Control (*n* = 40)	*p* value
Nausea				
0–48 h	17 (44)	19 (44)	23 (58)	0.369
0–6 h	16 (41)	16 (37)	18 (45)	0.711
6–24 h	5 (13)*	12 (28)	18 (45)	0.007
24–48 h	1 (3)	3 (7)	4 (10)	0.436
Vomiting				
0–48 h	5 (13)	7 (16)	9 (23)	0.512
0–6 h	5 (13)	6 (14)	8 (20)	0.635
6–24 h	2 (5)	4 (9)	7 (18)	0.197
24–48 h	1 (3)	0 (0)	0 (0)	0.320

Values are numbers of patients (percentage)

*p < 0.05 compared with group C

**Table 3:** Comparisons of the severity of nausea in the three groups

	Ramosetron (*n* = 39)	Ondansetron (*n* = 43)	Control (*n* = 40)	*p* value
0–6 h	2.9 ± 3.5	2.0 ± 3.2	2.5 ± 3.1	0.725
6–24 h	0.4 ± 1.3*	1.1 ± 2.5	1.7 ± 2.4	0.003
24–48 h	0.1 ± 0.5	0.2 ± 0.8	0.3 ± 1.3	0.416

Values are mean ± SD in parentheses using the VAS, where 0 indicates no nausea and 10 the worst imaginable nausea.

*p < 0.05 compared with group C

**Table 4:** Requirement for rescue antiemetics and the frequency of complete response to administrated rescue antiemetics

	Ramosetron (*n* = 39)	Ondansetron (*n* = 43)	Control (*n* = 40)	*P* value
Rescue antiemetics	9 (23)*	14 (33)	20 (50)	0.039
0–6 h	9 (23)	10 (23)	15 (38)	0.253
6–24 h	4 (10)	8 (19)	12 (30)	0.085
24–48 h	1 (3)	1 (2)	2 (5)	0.840
Complete response	37 (95)	39 (91)	36 (90)	0.772
0–6 h	25 (64)	24 (56)	21 (53)	0.562
6–24 h	32 (82)	32 (74)	20 (50)^†^	0.005
24–48 h	37 (95)	39 (91)	35 (88)	0.534

Values are numbers of patients (percentage)

The complete response was defined as no additional postoperative nausea and vomiting nor the requirement for rescue antiemetics

*p < 0.05 compared with group C

^†^*p* < 0.05 compared with the other two groups

**Table 5:** Comparisons of pain level and requirement for rescue pain killer in the three groups

	Ramosetron (*n* = 39)	Ondansetron (*n* = 43)	Control (*n* = 40)	*P* value
Pain score (VAS)				
0–6 h	5.3 ± 2.3	3.5 ± 1.9*	5.0 ± 2.5	0.001
6–24 h	4.4 ± 2.4	3.7 ± 1.9	4.6 ± 2.7	0.215
24–48 h	3.4 ± 2.6	2.9 ± 2.4	2.9 ± 2.6	0.081
Rescue pain killer	33 (85)	33 (77)	34 (85)	0.541
0–6 h	21 (54)	17 (40)	25 (63)	0.106
6–24 h	20 (51)	24 (56)	24 (60)	0.738
24–48 h	16 (41)	8 (19)	14 (35)	0.074

Values are numbers of patients (percentage) or mean ± SD in parentheses; pain scores were assessed using the VAS (0 indicates no pain and 10 the worst imaginable pain)

*p < 0.05 compared with the other two groups

**Table 6:** Postoperative adverse effects

	Ramosetron (*n* = 39)	Ondansetron (*n* = 43)	Control (*n* = 40)	*p* value
Headache	10 (26)	12 (28)	4 (10)	0.100
0–6 h	7 (18)	9 (21)	1 (3)*	0.036
6–24 h	6 (15)	7 (16)	3 (8)	0.436
24–48 h	2 (5)	1 (2)	2 (5)	0.738
Dizziness	16 (41)	13 (30)	16 (40)	0.530
0–6 h	15 (39)	12 (28)	14 (35)	0.585
6–24 h	8 (42)	7 (16)	4 (10)	0.431
24–48 h	4 (10)	2 (5)	3 (8)	0.567
Drowsiness	16 (41)	15 (35)	15 (38)	0.848
0–6 h	14 (36)	13 (30)	12 (30)	0.816
6–24 h	3 (8)	5 (12)	5 (13)	0.814
24–48 h	4 (10)	1 (2)	1 (3)	0.281

Values are numbers of patients (percentage)

*p < 0.05 compared with the other two groups

## Discussion

The most important finding of this study was that the incidence and severity of postoperative nausea were reduced effectively in the ramosetron group during the 6- to 24-h period compared with the control group after arthroscopic rotator cuff repair. The requirement for rescue antiemetics during 0- to 48-h period in the ramosetron group was significantly less than in the control group. Additionally, the frequency of complete response to administered rescue antiemetics in the ramosetron group and ondansetron group were significantly higher than that in the control group.

Rotator cuff repair can potentially cause severe postoperative pain. Although arthroscopic rotator cuff repair is minimally invasive, severe pain during the first several days after surgery is common. Various methods, including injection or infusion of local analgesics, regional nerve block, and IV-PCA, have been proposed to effectively reduce postoperative pain.

Current trends in the use of multimodal analgesia after surgery are increasingly popular to preventing postoperative pain. It involves administering a combination of opioid and nonopioid analgesics before, during, and after surgery that act at different sites within the central and peripheral nervous systems in an effort to improve pain control while eliminating opioid-related adverse effects. However, a combination of anesthetic and analgesic agents commonly provokes PONV [21, 29, 30], and multimodal pain management after surgery remains a challenging issue. Especially, IV-PCA opioid among multimodal analgesia facilitates pain management, early ambulation, reduces the length of hospital stay, and improves postoperative outcome. At the same time, however, it is frequently accompanied by critical complications, such as PONV.

Ramosetron is a newly developed 5-HT3 antagonist with a higher affinity and longer duration of action than that of the previously developed 5-HT3 antagonist such as ondansetron and granisetron. Several previous studies have reported that ramosetron is superior to ondansetron in preventing vomiting and reducing severity of nausea after surgery [6, 31]. Ramosetron has a significantly higher binding affinity for 5-HT3 receptors and a slower receptor-dissociation rate than the conventional 5-HT3 receptor antagonist ondansetron, resulting in more potent and longer-acting receptor-blocking effects. The elimination half-life of ramosetron (5.8 ±1.2h) is longer than that of ondansetron (3.8±1.0h) [32]. Ramosetron, another selective 5-HT-3 receptor, is involved in nociceptive pathways and binds to opioid μreceptors exhibiting agonist activity, resulting in a peripheral antinociceptive effect [33, 34]. Descending serotonergic neurons from the rostral ventromedial medulla facilitate nociceptive signaling in models of cancer-induced bone pain, inflammatory pain, and neuropathic pain [35].

Previous investigations have shown that 0.3 mg of ramosetron was more effective than 4 mg of ondansetron in patients with spine surgery, total knee arthroplasty, and laparoscopic cholecystectomy [6, 22, 31] and as effective as 8 mg of ondansetron in patients with gynecological surgery and laparoscopic surgery [28]. However, these studies have presented limited results, as they failed to have a control group; in particular, the study on PONV prevention after arthroscopic rotator cuff repair has not been yet. To our knowledge, ours is the first study to evaluate the effectiveness of ramosetron in surgery of arthroscopic rotator cuff repair.

Notably, ramosetron was superior to ondansetron and placebo in preventing PONV and improving complete response in the 6- to 24-h period in the present study. PONV often occurs between 12 and 24 h after surgery due to several factors, including food intake after prolonged preoperative fasting, early ambulation, use of opiates for pain control, reduced effectiveness of intraoperatively administered antiemetics, or residual anesthetics [36]. After 24 h, the emetic events in all groups were markedly reduced. In this regard, it may be questionable whether antiemetic agents are needed to prevent PONV after 24 h. However, arthroscopic procedures can still cause severe postoperative pain requiring considerable amount of opioid, especially during the first 24–48 h after rotator cuff repairs [1]. Therefore, it is reasonable to use ramosetron, which can reduce PONV by 48 h postoperatively.

5-HT3 antagonists (ramosetron and ondansetron) are reported to have side effects including headache, dizziness, and drowsiness [37]. In our study, the incidence of headache was significantly higher in patients receiving 5-HT3 antagonists than in patients of control group by 0–6 h postoperatively, but the incidences of headache by 6–48 h and dizziness and drowsiness did not differ for the three groups. More RCTs would be required to reach a firm conclusion regarding the comparative incidence of side effects of ramosetron and ondansetron.

### Future works

Although ramosetron and ondansetron can reduce nausea within 24 h, there was no significant difference in vomiting. This may mean that ramosetron cannot control other factors involved in vomiting. The effect of other factors such as histamine, muscarinic, and dopamine receptors on vomiting is greater than that of serotonin receptors. Further discussion on this issue will be needed in the future.

This study had several limitations. First, our study population consisted of patients with different severities of rotator cuff tear. Second, our data cannot explain why the ability to control pain management is superior in the ondansetron group. Because a good therapeutic effect of PONV can lead to increase amount of opioid use, this may reduce pain level. However, there were no differences in opioid consumption, and pain management was slightly better in the ondansetron group. The control of PONV does not influence pain level in our data. In addition, this study included a small number of cases. Further, pain was simply measured in accordance with each postoperative period without distinguishing non-resting pain and resting pain. Since the degree of pain may differ according to the activity of the individual, it should be taken into account when assessing the severity of pain.

## Conclusion

Ramosetron use led to a lower incidence, mild severity of nausea, and reduced use of rescue antiemetic drug after arthroscopic rotator cuff repair during the 6- to 24-h postoperative period than the control.

## Data Availability

All data generated during this study are included in this published article [and its supplementary information files].

## Abbreviation

PONV: Postoperative nausea and vomiting
